# Supplementary material for: ESR1 mutant breast cancers show elevated basal cytokeratins and immune activation
Source: Nat Commun. 2022 Apr 19;13:2011. doi: 10.1038/s41467-022-29498-9 (PMC9019037; doi:10.1038/s41467-022-29498-9)
Supplement: Supplementary file 1 — Supplementary Information File [file 41467_2022_29498_MOESM1_ESM.pdf]

Supplementary Figure 1

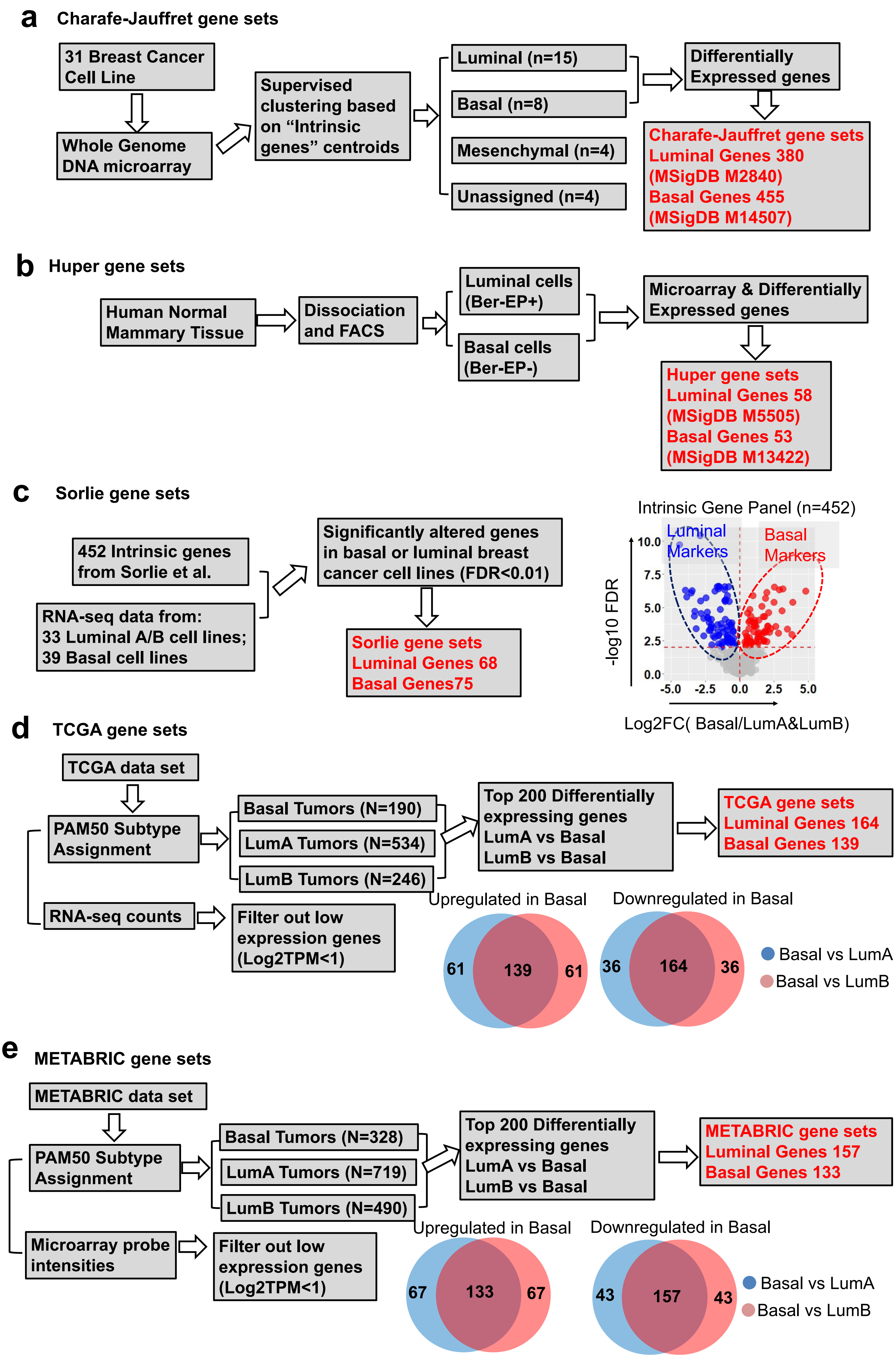

Supplementary Figure 1. Schematic flow charts of the generation process of the four pairs of luminal/basal gene sets. (Related to Fig. 1)

a) Charafe-Jauffret gene set was obtained based on a microarray data of 31 breast cancer cell lines after supervised clustering and subtype assignment. Differentially expressed genes from 15 luminal and 8 basal breast cancer cells derived 380 and 455 luminal and basal genes.

b) Huper gene set was computed based on microarray data of flow cytometry sorted basal and luminal epithelial cells from a normal human mammary tissue. This gene set contains 58 luminal and 53 basal genes.

c) Sorlie gene set was derived based on the differentially expressed genes between 39 basal and 33 luminal breast cancer cell lines among the 452 intrinsic gene panel reported by Sorlie et al. The volcano plot on the right panel represents the differentially gene expression distribution of the 452 genes, genes with FDR<0.01 were highlighted and used for the gene sets.

d) For the TCGA gene set, differentially expressed genes (FDR<0.01) were called between basal and luminal A or basal and luminal B ER+ tumors using raw counts after filtering out low expression genes (maximum Log2 TPM expression across all samples <1). The top 200 increased genes from these two comparisons were further intersected (Venn diagram). Shared upregulated (n=139) or downregulated (n=164) DE genes between basal-to-LumA and basal-to-LumB comparisons tumors were called as TCGA gene sets.

e) For the METABRIC gene set, differentially expressed genes (FDR<0.01) were called between basal and luminal A or basal and luminal B ER+ tumors using normalized probe intensities of each gene. The top 200 increased genes from these two comparisons were further intersected (Venn diagram). Shared upregulated (n=133) or downregulated (n=157) DE genes between basal-to-LumA and basal-to-LumB comparisons tumors were called as METABRIC gene sets.

Supplementary Figure 2

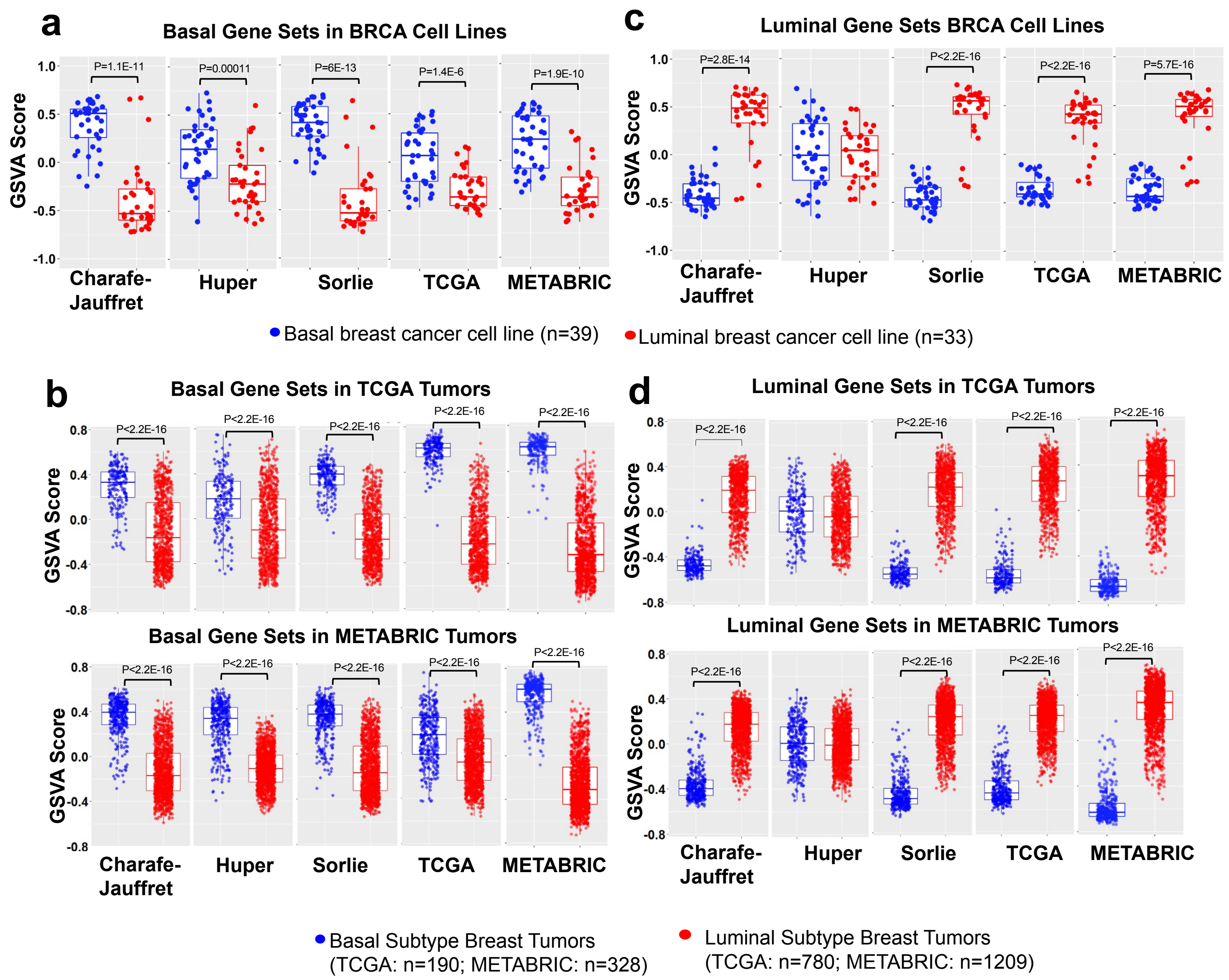

Supplementary Figure 2. Quality control of the four pairs of luminal/basal gene sets in breast cancer cell lines and tumors. (Related to Fig. 1)

a) and c) Dot plots showing GSVA score of the five pairs of basal (a) /luminal (c) gene sets enrichment in luminal (n=33) and basal (n=39) breast cancer cells as quality controls. Each plot represents mean ± SD from GSVA score. Box plots span the upper quartile (upper limit), median (centre) and lower quartile (lower limit). Whiskers extend a maximum of 1.5X IQR. Mann Whitney U test was used within each group (** p<0.01).

b) and d) Box plots showing GSVA score of the five pairs of basal (b) /luminal (d) gene sets enrichment in luminal and basal breast cancer tumors from TCGA (n=190 basal, n=780 luminal) and METABRIC (n=328 basal, n=1209 luminal) as quality controls. Box plots span the upper quartile (upper limit), median (centre) and lower quartile (lower limit). Whiskers extend a maximum of 1.5X IQR. Mann Whitney U test (two-sided) was used within each group.

Source data are provided as a Source Data file for a-d.

Supplementary Figure 3

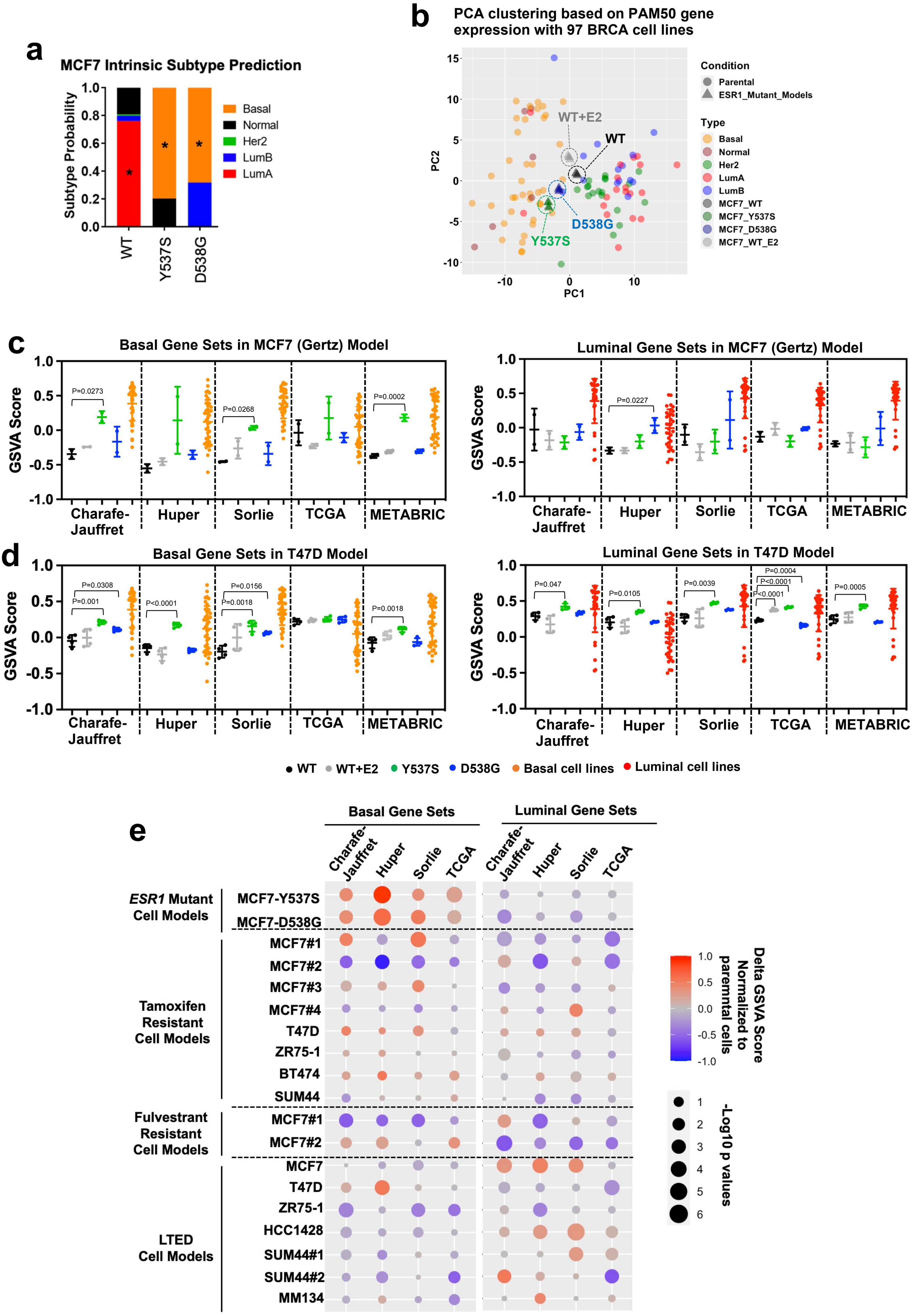

Supplementary Figure 3. *ESR1* mutant cell models but not other endocrine resistant models show increased basal marker enrichment. (Related to Fig. 1)

a) Stacked plot shows probability distribution of five breast cancer subtypes in MCF7 ESR1 WT and mutant cells. The dominant subtype called by genefu is indicated with asterisk symbols.

b) PCA plot showing the clustering of ESR1 WT and mutant cells with other 97 characterized breast cancer cell lines.

c) and d) Dot plots showing GSVA score of the five pairs of basal/luminal marker gene sets enrichment in MCF7 (Gertz) (c) and T47D (d) genome-edited ESR1 mutant cell models. Two individual clones were used as biological replicates for Gertz model, while four biological replicates were used for T47D cell model. Data are presented as mean ± SD. Enrichment scores in luminal (n=33) and basal (n=39) breast cancer cell lines were used as positive controls. Dunnett's test (two-sided) was used within each group.

e) Graphic view of delta GSVA enrichment score of 19 different endocrine resistant breast cancer models normalized to their corresponding parental cell counterparts. Color scale represents changes of enrichment scores and dot size shows significance. Data sets were downloaded and analyzed from GEO. (Tamoxifen resistant models: GSE128458, GSE104985, GSE111151, GSE26459 and GSE106681; Fulvestrant resistant models: GSE118713 and GSE104985; LTED models: GSE75971 and GSE116744). Detailed sources information can be found in Supplementary Table 8.

Source data are provided as a Source Data file for a-e.

Supplementary Figure 4

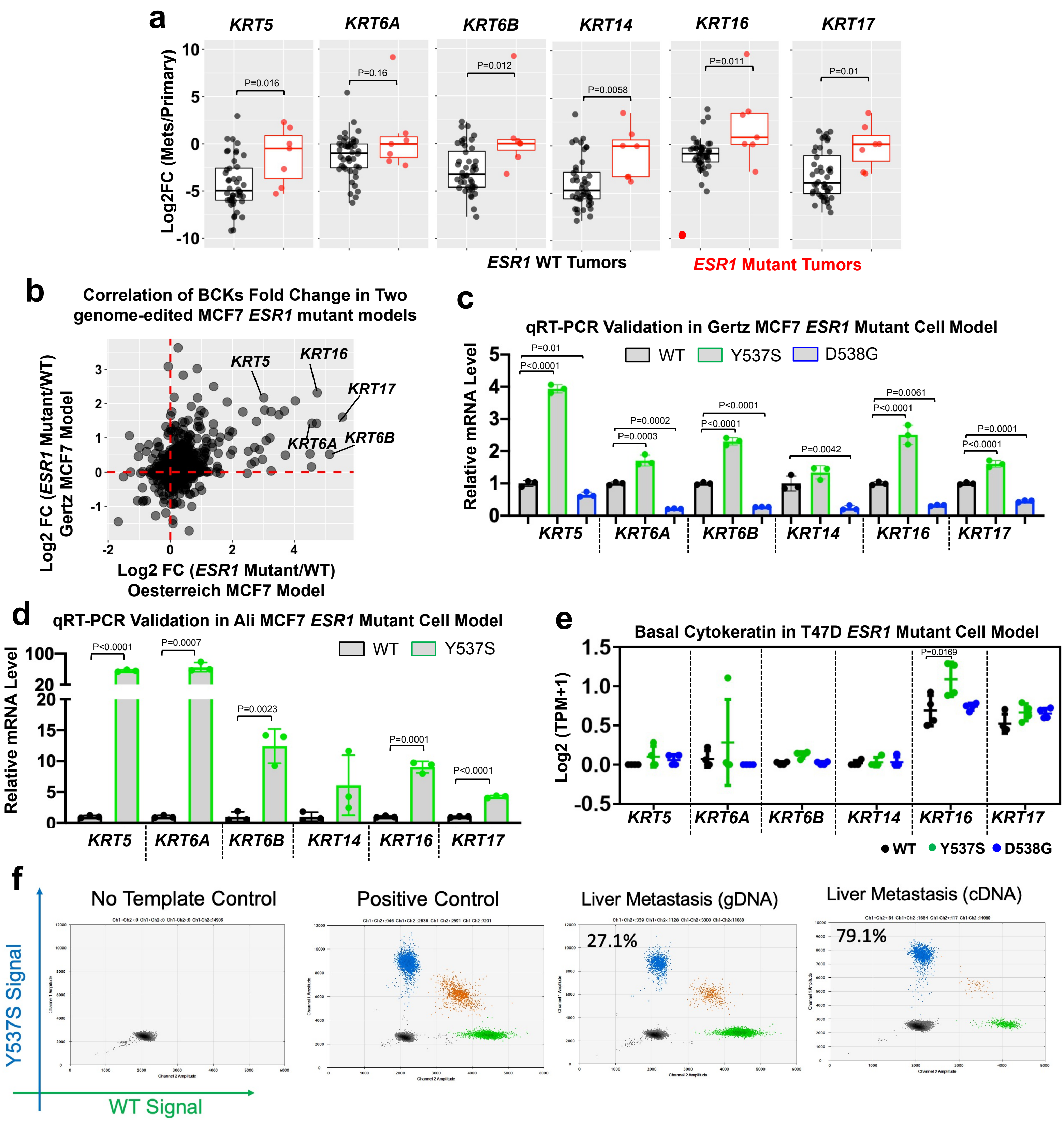

Supplementary Figure 4. Basal cytokeratins are increased in other genome-edited *ESR1* mutant cell models. (Related to Fig. 2)

a) Box plots representing the six basal cytokeratin expression in primary-matched paired metastatic tumor samples. Log2 (CPM+1) values were used for calculation. Expressional fold changes in each metastatic tumor were normalized to the matched primary tumor. Box plots span the upper quartile (upper limit), median (centre) and lower quartile (lower limit). Whiskers extend a maximum of 1.5X IQR. Mann-Whitney U test (two-sided) was performed to compare the expression between *ESR1* WT (N=44) or *ESR1* mutant (N=7) paired tumors. (* $p<0.05$; ** $p<0.01$)

b) Two-dimensional plot showing the correlation of basal marker gene fold changes in Oesterreich and Gertz MCF7 genome-edited Y537S/D538G cells (normalized to WT vehicle group). Increased basal cytokeratins were labelled with gene names.

c) and d) Bar graphs representing qRT-PCR measurement of *KRT5/6A/6B/14/16/17* mRNA levels in the Gertz (c) and Ali (d) MCF7 WT and *ESR1* mutant cells. ΔΔCt method was used to analyze relative mRNA fold changes normalized to WT cells and *RPLP0* levels were measured as the internal control. Each bar represents mean ± SD with three biological replicates. These experiments were done once for each. Dunnett's test (c) (two-sided) and student's t test (d) (two-sided) were used to compare BCKs expression levels between WT and mutants respectively.

e) Dot plot representing all six basal cytokeratins expression in the Oesterreich T47D *ESR1* mutant cell models. Each dots represent the Log2 (TPM+1) value from the original RNA-seq data set (GSE89888) with n=4 biologically independent replicates. Dunnett's test (two-sided) was applied.

f) Scattered plot representing Y537S mutation identification from droplet digital PCR on the ER+ liver metastasis at DNA and RNA levels. Water and Y537S mutant cell model DNA were used as positive and negative controls.

Source data are provided as a Source Data file for a-e.

Supplementary Figure 5

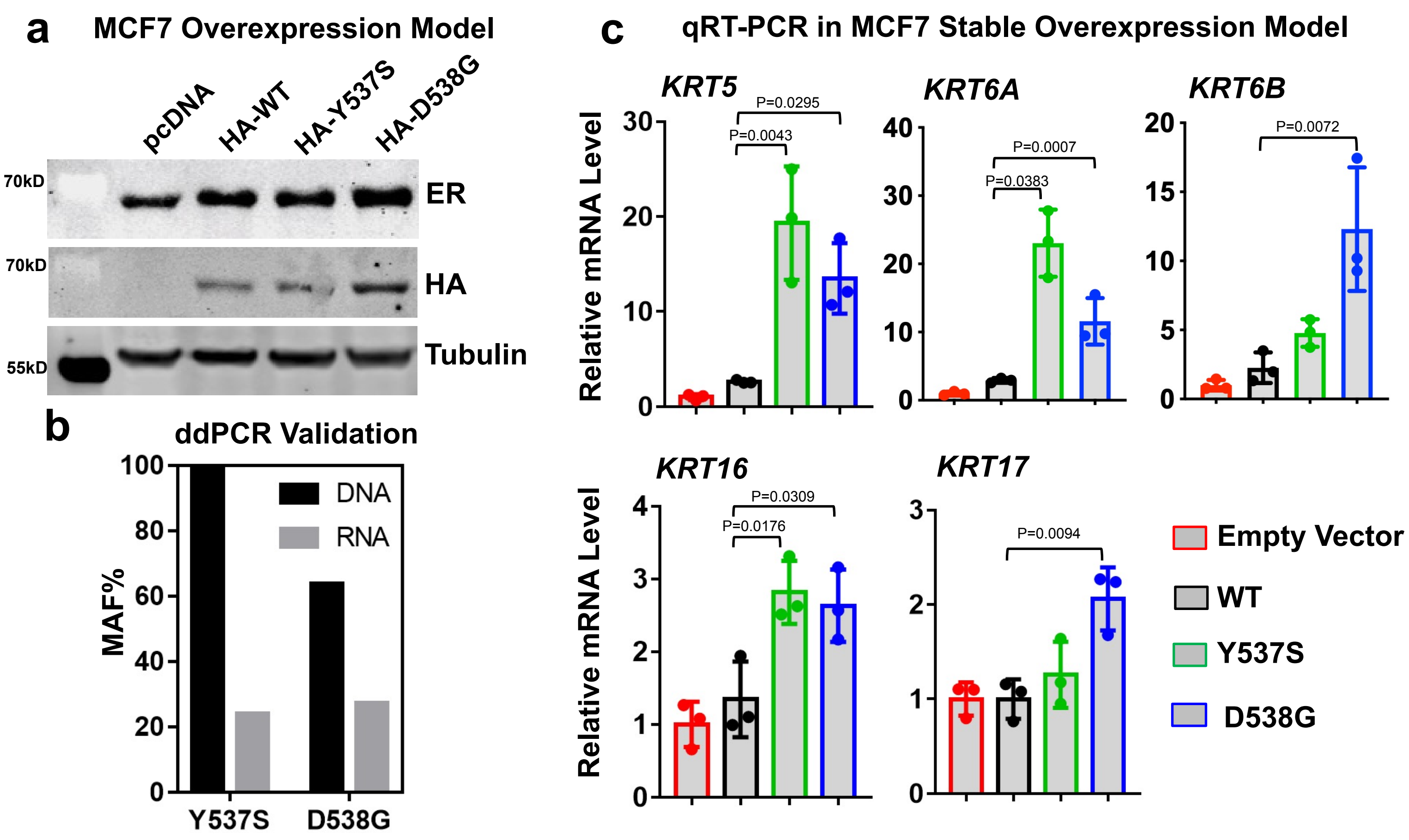

Supplementary Figure 5. Basal cytokeratins are increased in stably overexpressed *ESR1* mutant cell models. (Related to Fig.2)

a) Immunoblot validation of MCF7 *ESR1* mutant overexpression cells. Total ER and overexpressed ER (HA-tagged) were detected and tubulin was used as loading control. This experiment was done once. Uncropped images are provided in Supplementary Figure 13.

b) ddPCR validation of mutation allele frequencies in Y537S and D538G overexpression cell model at both DNA (gDNA) and RNA (cDNA) levels. This experiment was done once.

c) Bar graphs representing qRT-PCR measurement of *KRT5/6A/6B/16/17* mRNA levels in MCF7 overexpression *ESR1* mutant cell models. ΔΔCt method was used to analyze relative mRNA fold changes normalized to empty vector cells and *RPLP0* levels were measured as the internal control. Each bar represents mean ± SD with three biological replicates. Data were from one representative experiment of two independent repeats. Dunnett’s test (two-sided) was used to compare the gene expression of each *ESR1* mutant group to WT cells.

Source data are provided as a Source Data file for b,c.

Supplementary Figure 6

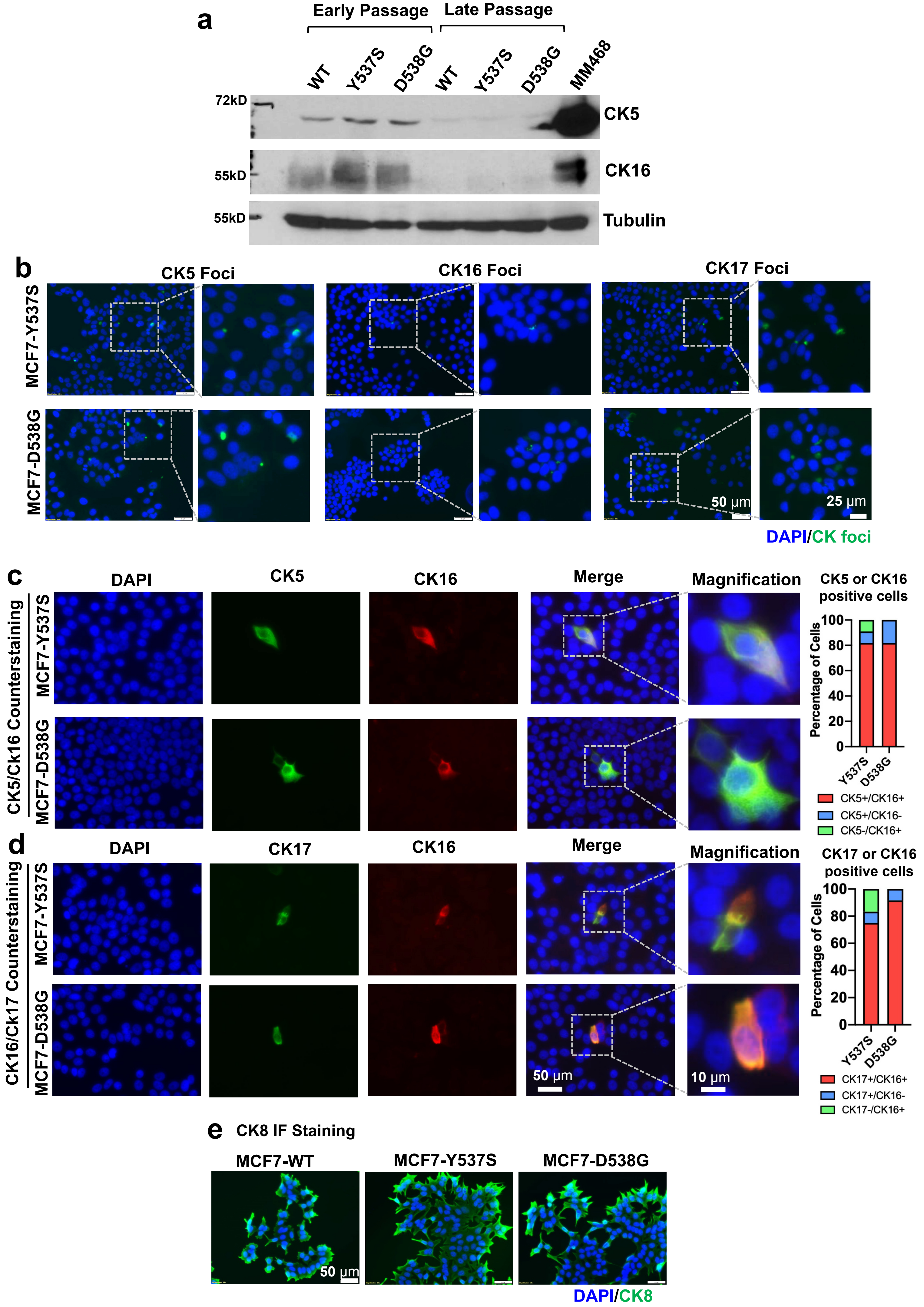

Supplementary Figure 6. Basal cytokeatins are heterogeneously expressed in *ESR1* mutant cells. (Related to Figure 2)

a) Immunoblot validation of CK5 and CK16 expression MCF7 *ESR1* mutant cells in low (P6-P8) and high (P30-P32) passages. Tubulin was used as loading control. This experiment was done once. Uncropped images are provided in Supplementary Figure 13.

b) Representative images of CK5/16/17 foci in MCF7 *ESR1* mutant cells. Images were taken under 20x magnification. Specific regions with foci detected were further zoomed in as magnified views. Data were from one representative of three independent replicates.

c) and d) Left panel: Representative images of CK5/CK16 (c) and CK5/CK17 (d) counterstaining in MCF7 *ESR1* mutant cells. Images were taken under 20x magnification. Specific regions with CK+ cells were further zoomed in as magnified views. Right panel: Stacked plots representing the quantification of percentage of cells with double CK subtype positivity (red) or single CK subtype positivity (blue/green) among all CK+ cells (n=11 for CK5/CK16 co-staining quantification, n=12 for CK16/CK17 co-staining quantification). This experiment was done once.

e) Representative images of luminal cytokeratin CK8 staining in MCF7 WT and *ESR1* mutant cells. Images were taken under 20x magnification. This experiment was done once.

Source data are provided as a Source Data file for c,d.

Supplementary Figure 7

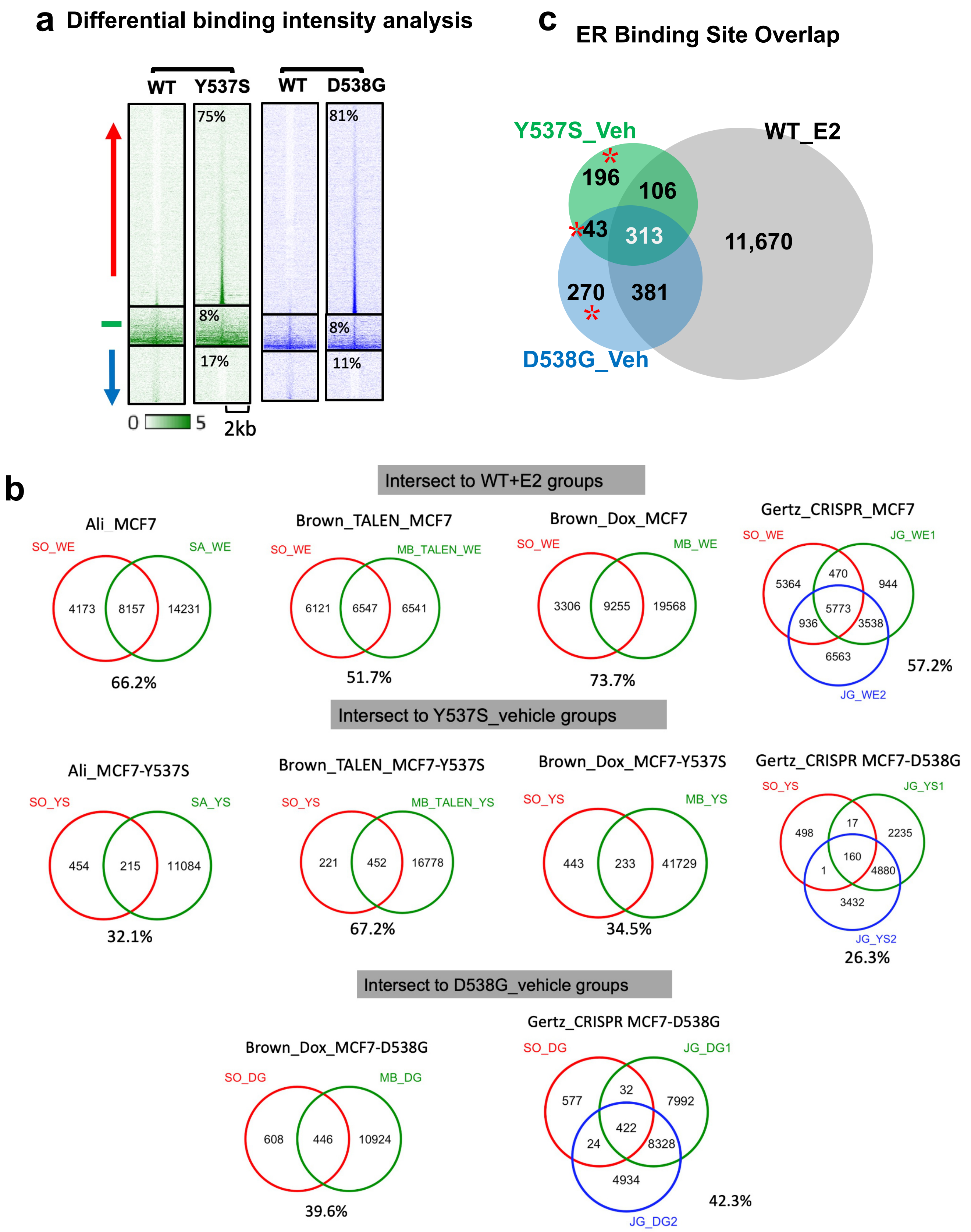

Supplementary Figure 7-Continued

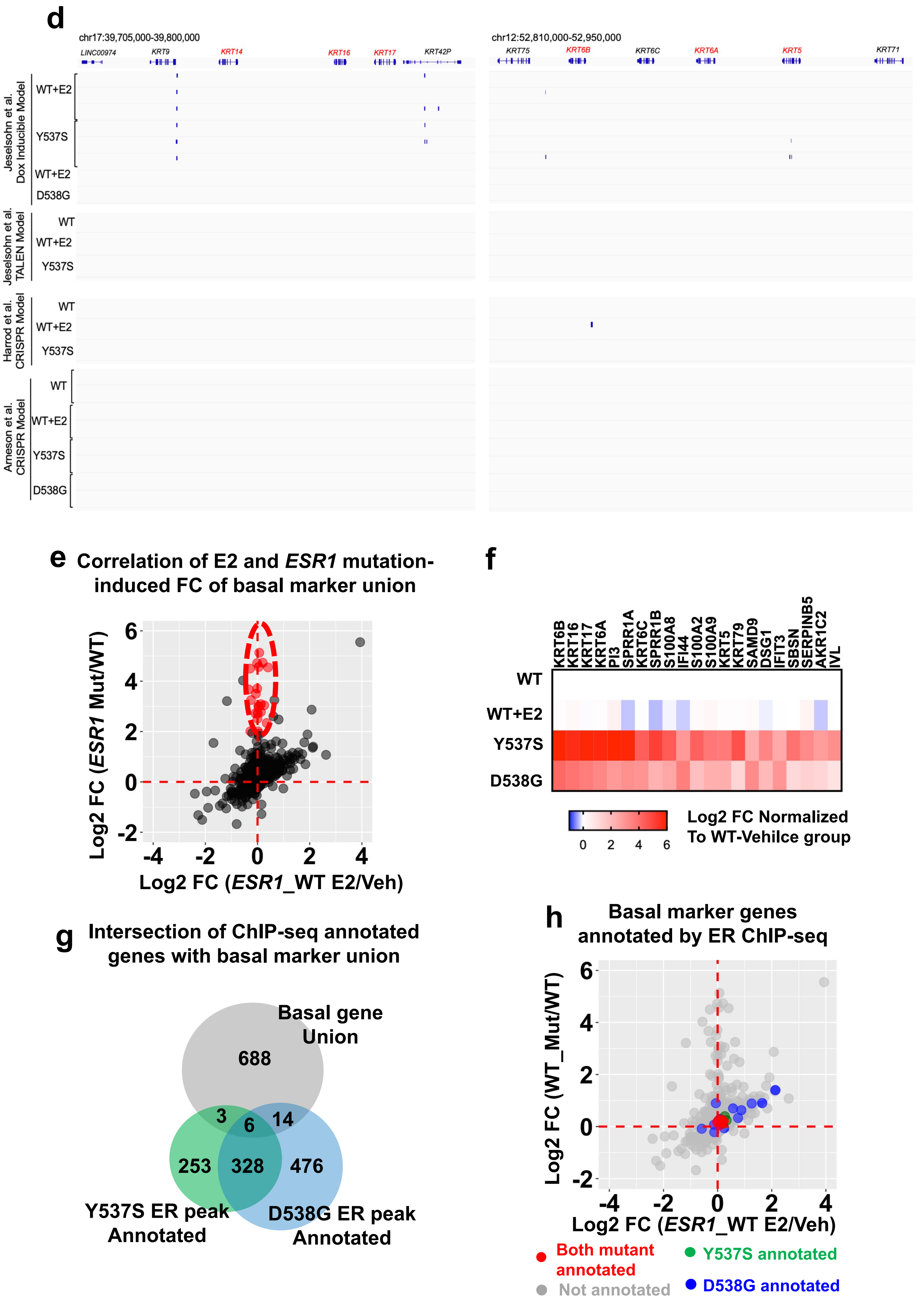

Supplementary Figure 7. ChIP-seq profiling of mutant ER cistrome reveals limited regulation on basal marker gene from ER genomic binding. (Related to Fig.3)

a) Heatmaps of differential ER binding intensities in Y537S, D538G mutants compared to WT ER in a pairwise manner, shown in a horizontal window of ± 2kb from the peak center. The pairwise comparison between WT and mutant samples were performed to calculate the fold change (FC) of intensities and the binding sites were sub-classified into sites with increased intensity (FC>2, red arrow), decreased intensity (FC<-2, blue arrow), and non-changed intensity (-0.2<FC<0.2, green line). Percentage of each subgroups were labelled on the heatmaps respectively.

b) Venn diagrams representing the intersection of ER ChIP-seq used in the study to other three independent data sets from Harrod et al. (Ali model, GSE78286), Jeselsohn et al. (Brown model, GSE94493) and Arneson et al. (Gertz model, GSE148279) ChIP-seq profile from the three different conditions were analyzed including *ESR1* WT+E2, Y537S and D538G mutant cells. Peak overlapping percentages of ChIP-seq from this study were indicated below each diagram.

c) Venn Diagrams showing the occupancy intersection between WT-E2, Y537S-vehicle and D538G-vehicle groups in MCF7 cell model. *De novo* mutant ER peaks were labelled with asterisk symbols.

d) Genomic track showing called ER binding peaks at KRT14/16/17 (left panel) and KRT5/6A/6B (right panel) loci from four different ER ChIP-seq data sets of MCF7 *ESR1* mutant cells from Harrod et al. (Ali model, GSE78286), Jeselsohn et al. (Brown model, GSE94493) and Arneson et al. (Gertz model, GSE148279).

e) Scattered plot showing the correlation of basal gene fold changes of MCF7 *ESR1* mutant (average FC of Y537S and D538G) cells and E2 stimulation in WT cells. 21 skewed basal genes were highlighted in red (Log2FC>|2| in mutants and <|0.5| in WT-E2 group).

f) Heatmap representing the fold changes of the 21 unqiue *ESR1* mutant-regulated basal genes (highlighted in Figure 7C) in WT-E2 and two mutant groups normalized to WT-vehicle group using RNA-seq data (GSE89888).

g) Venn digram showing interaction of Y537S (n=577) and D538G (n=778) ER peak annotated genes to the union of basal genes (n=711) collection. Annotated genes were called within -/+ 50 kb range of each ER peak.

h) Scattered plot showing the correlation of basal marker gene fold changes of MCF7 *ESR1* mutant (average FC of Y537S and D538G) cells and E2 stimulation in WT cells. Genes that were annotated by ER ChIP-seq data in Y537S (green), D538G (blue) and both mutants (red) were labelled accordingly.
Source data are provided as a Source Data file for e,f,h.

Supplementary Figure 8

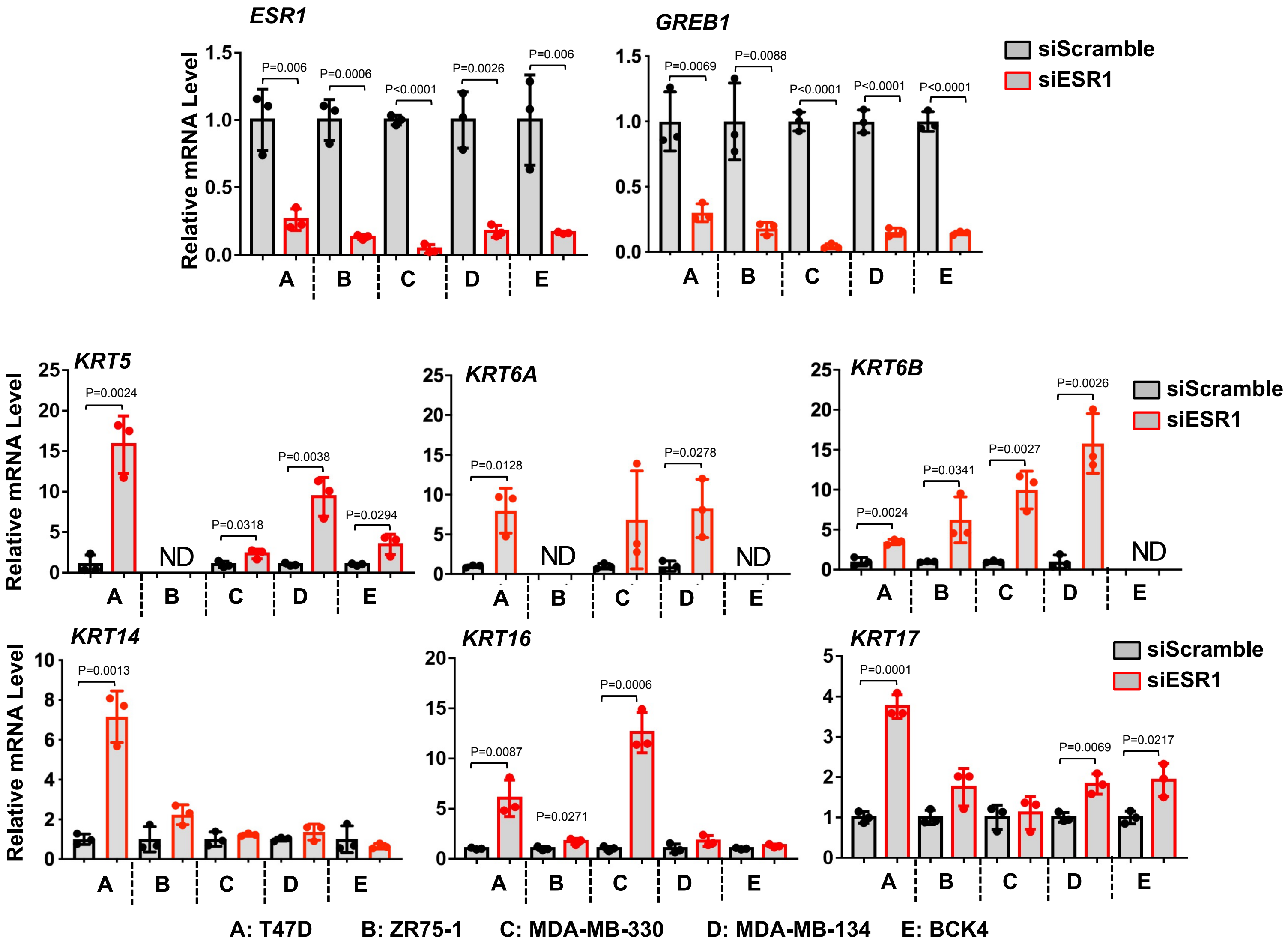

Supplementary Figure 8. Knockdown of *ESR1* generally increased basal cytokeratin expression in ER+ breast cancer cell lines. (Related to Fig. 3)
Bar graphs representing qRT-PCR measurement of *ESR1*, *GREB1* and *KRT5/6A/6B/14/16/17* mRNA levels in five ER+ breast cancer cells with siRNA knockdown of *ESR1* for 7 days. ΔΔCt method was used to analyze relative mRNA fold changes normalized to each siScramble groups and *RPLP0* levels were measured as the internal control. Each bar represents mean ± SD with three biological replicates. Student's test (two-sided) was used to compare the gene expression between scramble and knockdown groups. This experiment was done once. Genes with undetectable values were indicated as ND.
Source data are provided as a Source Data file.

Supplementary Figure 9

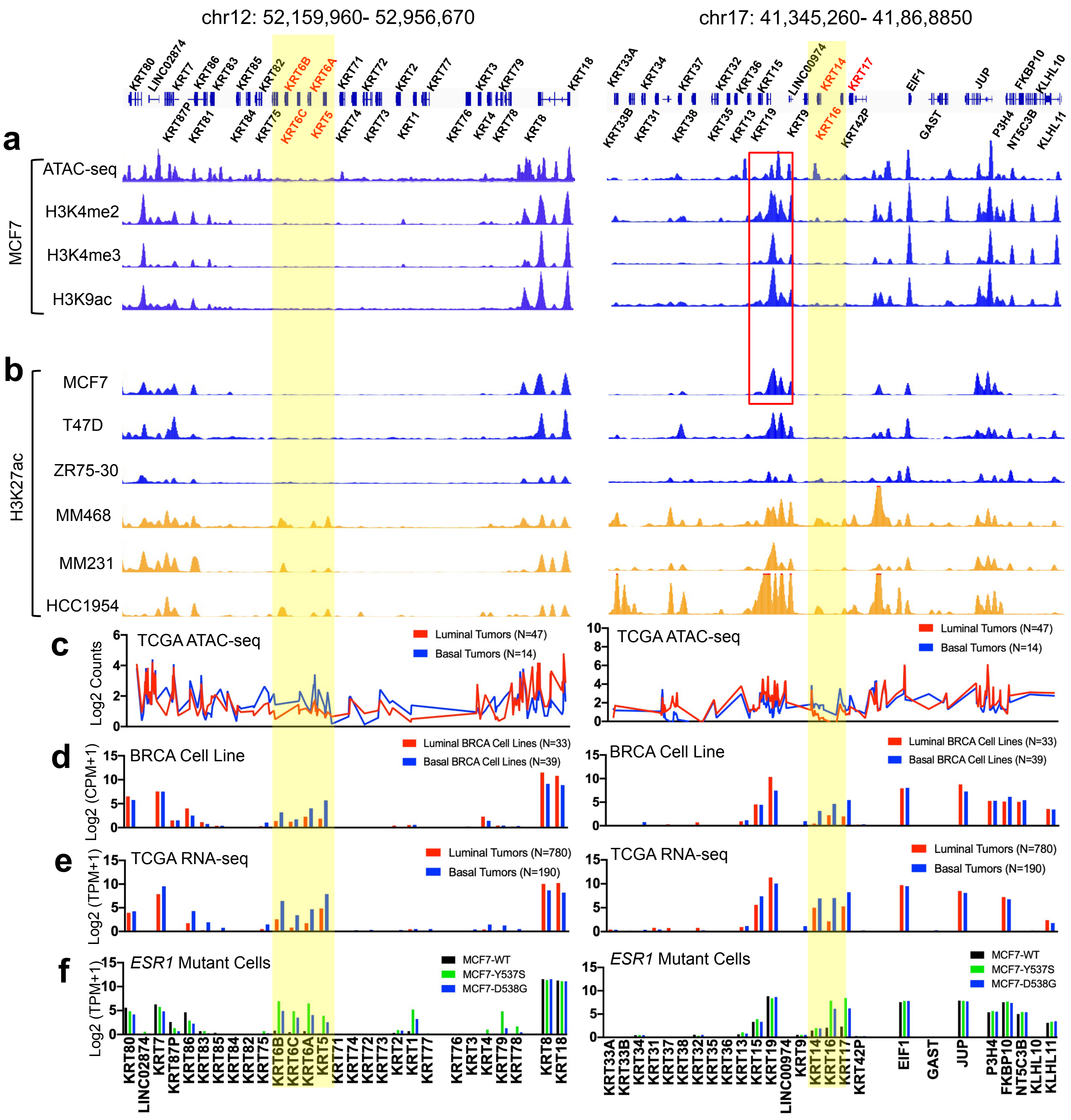

Supplementary Figure 9. Epigenetic landscapes of *KRT5/6A/6B* and *KRT14/16/17* loci in basal and luminal cells and tumors. (Related to Fig. 4)
Genomic track illustrations of *KRT5/6A/6B* and *KRT14/16/17* proximal regions with active histone modifications ChIP-seq (a) in MCF7 and H3K27 acetylation ChIP-seq in 3 luminal and 3 basal breast cancer cell lines (b). Data sets were visualized at WashU Genome Browser based on publicly available data sets from GEO (GSE102441, GSE69112, GSE65201 and GSE29069) and ENCODE (ENCSR875KOJ, ENCSR958MIB, ENCSR056UBA and ENCSR752UOD). Y axis represents the signal intensity of ChIP-seq data sets. TCGA ATAC-seq (c) and RNA-seq log2 (TPM+1) values (e) were compared between luminal and basal tumors with specific numbers labelled in the plots. In addition, proximal gene expressions were compared in 33 luminal and 39 basal breast cancer cell lines (d) and MCF7 *ESR1* mutant cell models (f) using Log2 (CPM+1) and Log2 (TPM+1) values respectively based on RNA-seq data. *KRT5/6A/6B* and *KRT14/16/17* loci were highlighted in light yellow. The super enhancer region featured by active histone modification at *KRT14/16/17* loci was highlighted in a red frame.
Source data are provided as a Source Data file for c-f.

Supplementary Figure 10

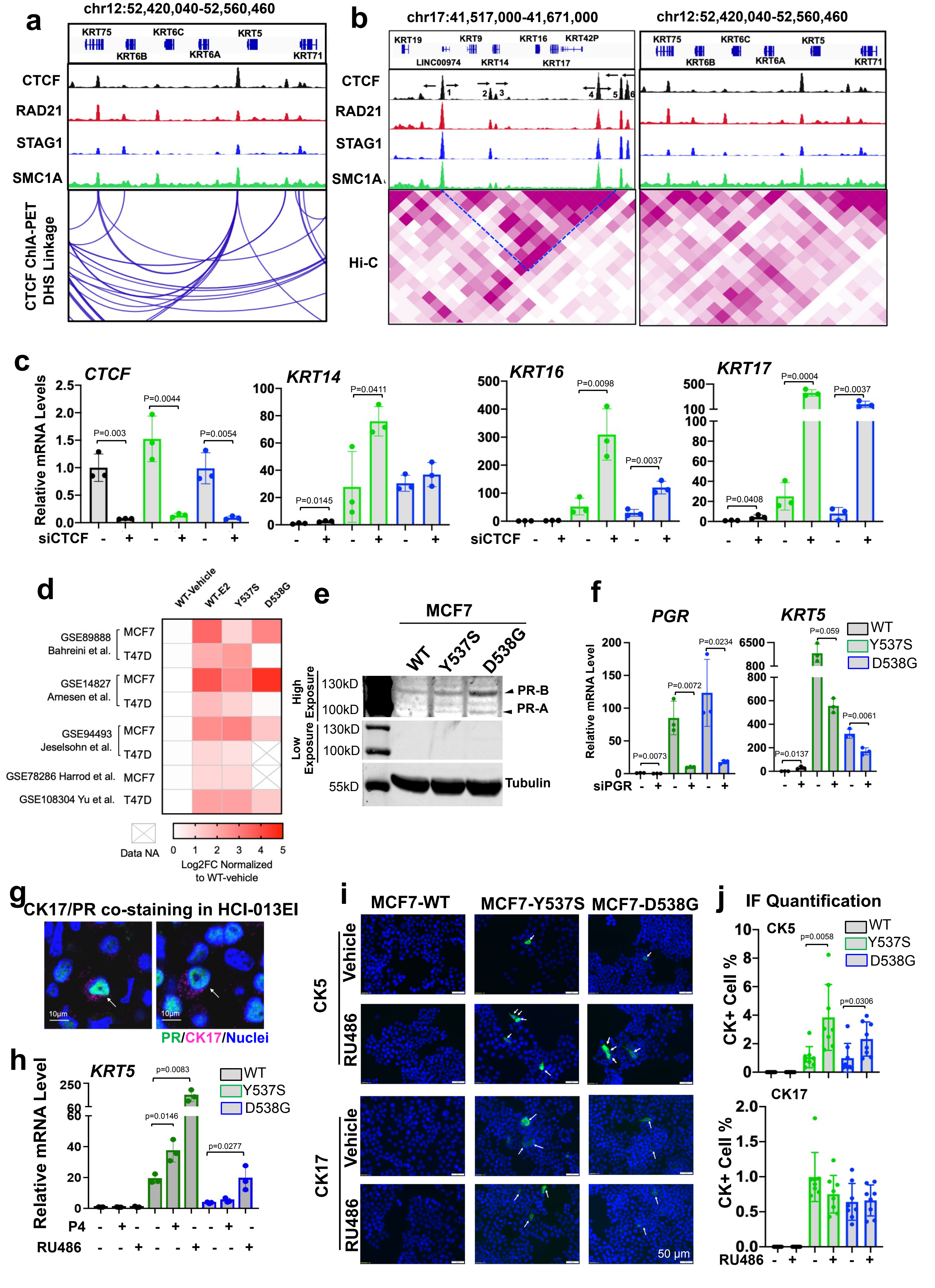

Supplementary Figure 10. BCKs are associated with a progesterone receptor binding enhancer orchestrated TAD in *ESR1* mutant cells. (Related to Fig.4)

a) Upper panel: Genomic track illustrating the CTCF/cohesion complex binding at *KRT5/6A/6B* proximal genomic region in MCF7 cells. CTCF and RAD21 ChIP-seq were downloaded from ENCODE (ENCSR560BUE and ENCSR703TNG). STAG1 and SMC1A ChIP-seq data were from GEO (GSE25021 and GSE76893). Y-axis represents signal intensity of each track. Include genomic coordinates. Lower panel: CTCF-driven chromatin loops visualized using a CTCF ChIA-PET data set in MCF7 cells (GSE72816) at the 3D Genome Browser platform. Each linkage represents a chromatin loop.

b) Genomic track illustrating CTCF/Cohesin complex binding and heatmap presentation of chromatin interaction scores at KRT14/16/17 and KRT5/6A/6B loci using a MCF7 Hi-C data set (GSE130916). Each bin represents a 10kb window. The interaction between CTCF peak#1 and #5 at KRT14/16/17 loci was highlighted with a blue frame.

c) Bar graphs showing qRT-PCR measurement of *CTCF*, *KRT14, 16* and *17* mRNA levels in MCF7 *ESR1* WT and mutant cells with siRNA knockdown of *CTCF* for 7 days. ΔΔCt method was used to analyze relative mRNA fold changes normalized to WT cells (siScramble group) and *RPLP0* levels were measured as the internal control. Each bar represents mean ± SD with three biological replicates. Student's test (two-sided) was used to compare the gene expression between scramble and knockdown groups. This experiment was done once.

d) Heatmap representing *PGR* gene expression fold change in WT+E2 and *ESR1* mutant groups from eight different *ESR1* mutant cell models with publicly available RNA-seq data sets.

e) Immunoblot detection PR in MCF7 *ESR1* mutant cells. Tubulin was used as a loading control. This experiment was done once. Uncropped images are provided in Supplementary Figure 13.

f) Bar graphs showing qRT-PCR measurement *PGR* and *KRT5* mRNA levels in MCF7 *ESR1* WT and mutant cells with siRNA knockdown of *PGR* for 7 days. Method was the same as described in c. Data is from one representative of three independent replicates. Student's test (two-sided) was used.

g) Representative images of CK17 and PR counter staining in *ESR1* mutant PDX model HCI-013EI. Images were taken under 20x magnification. This experiment was done once.
h) Bar graphs showing qRT-PCR measurement *KRT5* mRNA levels in MCF7 *ESR1* WT and mutant cells with 0.1% EtOH, 100 nM P4 and 1 μM RU486 treatment for 3 days. Method was the same as described in c. Data is from one representative of three independent replicates. Student's test (two-sided) was used
i) Representative images of immunofluorescence staining on CK5 and CK17 in MCF7 WT and *ESR1* mutant cells under 1 μM RU486 treatment for 3 days. Images were taken under 20x magnification. CK+ cells are pointed with white arrows.
j) Bar plots quantifying the percentages of CK positive cells of each group. Each bar represents mean ± SD from eight different regions combining from two independent experiments. Student's t test (two-sided) was used to compare BCKs positive cell percentage in the presence and absence of the treatment.
Source data are provided as a Source Data file for c,d,f,h,j.

Supplementary Figure 11

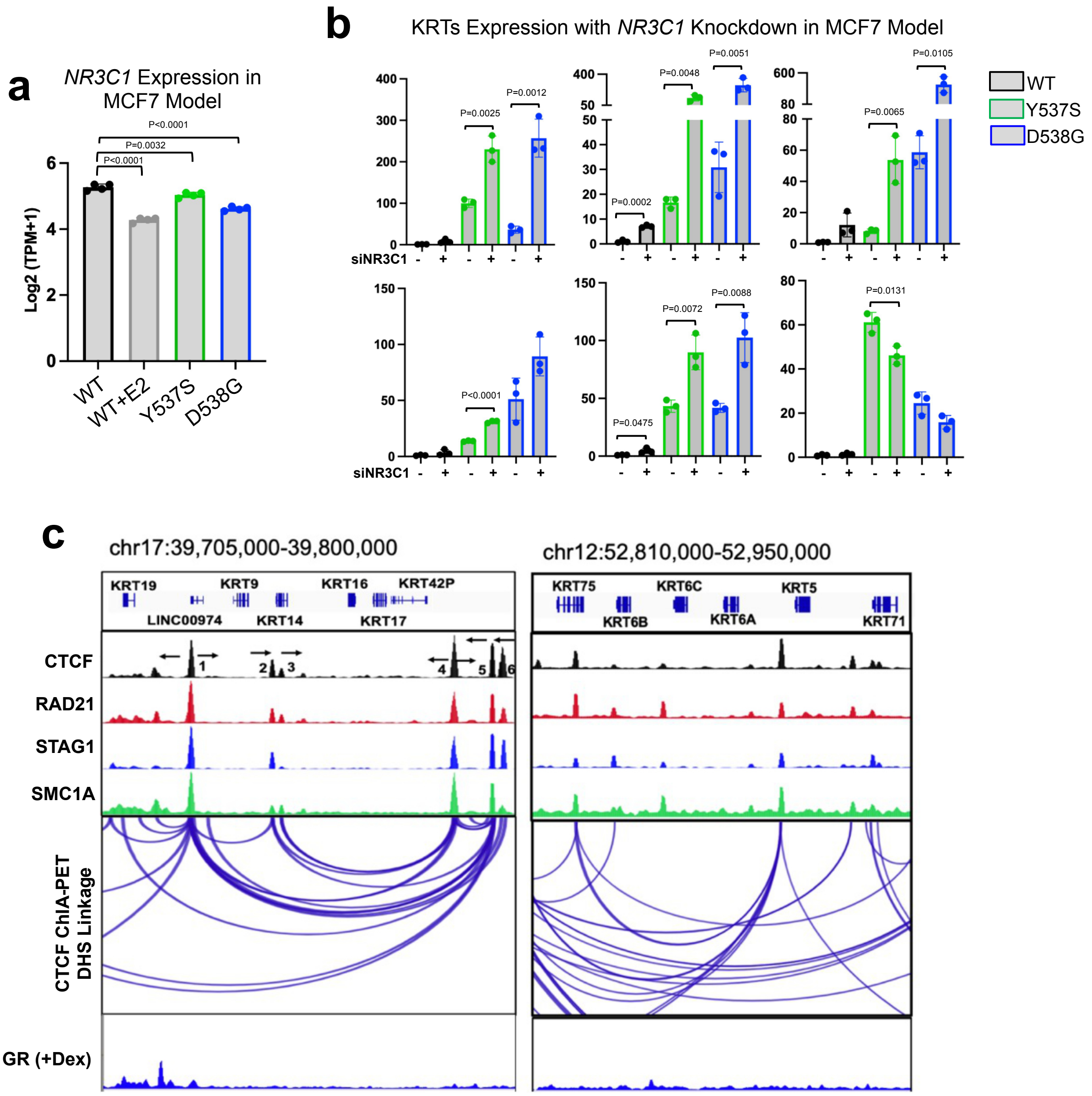

Supplementary Fig. 11. Glucocorticoid receptor is a suppressor of BCK expression in *ESR1* mutant cells. (Related to Fig.4)

a) Bar plot showing expression of *NR3C1* in MCF7 *ESR1* WT and mutant cells. Data were extracted from RNA-seq using log2(TPM+1) values with four biological replicates. Dunnett's test (two-sided) was used to compare each group versus WT vehicle group.

b) qRT-PCR measurement of *ESR1*, *KRT5/6A/6B/14/16/17* mRNA levels in MCF7 WT and *ESR1* mutant cells with NR3C1siRNA knockdown for 7 days. mRNA fold change normalized to WT cells; RPLP0 levels were measured as internal control. Each bar represents mean ± SD with three biological replicates. Data shown are representative from three independent experiments. Student's t-test (two-sided) was used to compare the gene expression between scramble and knockdown groups.

c) Genomic track illustrating the CTCF/Cohesion complex binding at *KRT14/16/17* and *KRT5/6A/6B* proximal genomic region in MCF7 cells. CTCF and RAD21 ChIP-seq were downloaded from ENCODE. STAG1 and SMC1A ChIP-seq data were from GEO (GSE25021 and GSE76893). GR ChIP-seq with dexamethasone treatment was downloaded from GSE72249. Y-axis represents signal intensity of each track. Source data are provided as a Source Data file for a,b.

Supplementary Figure 12

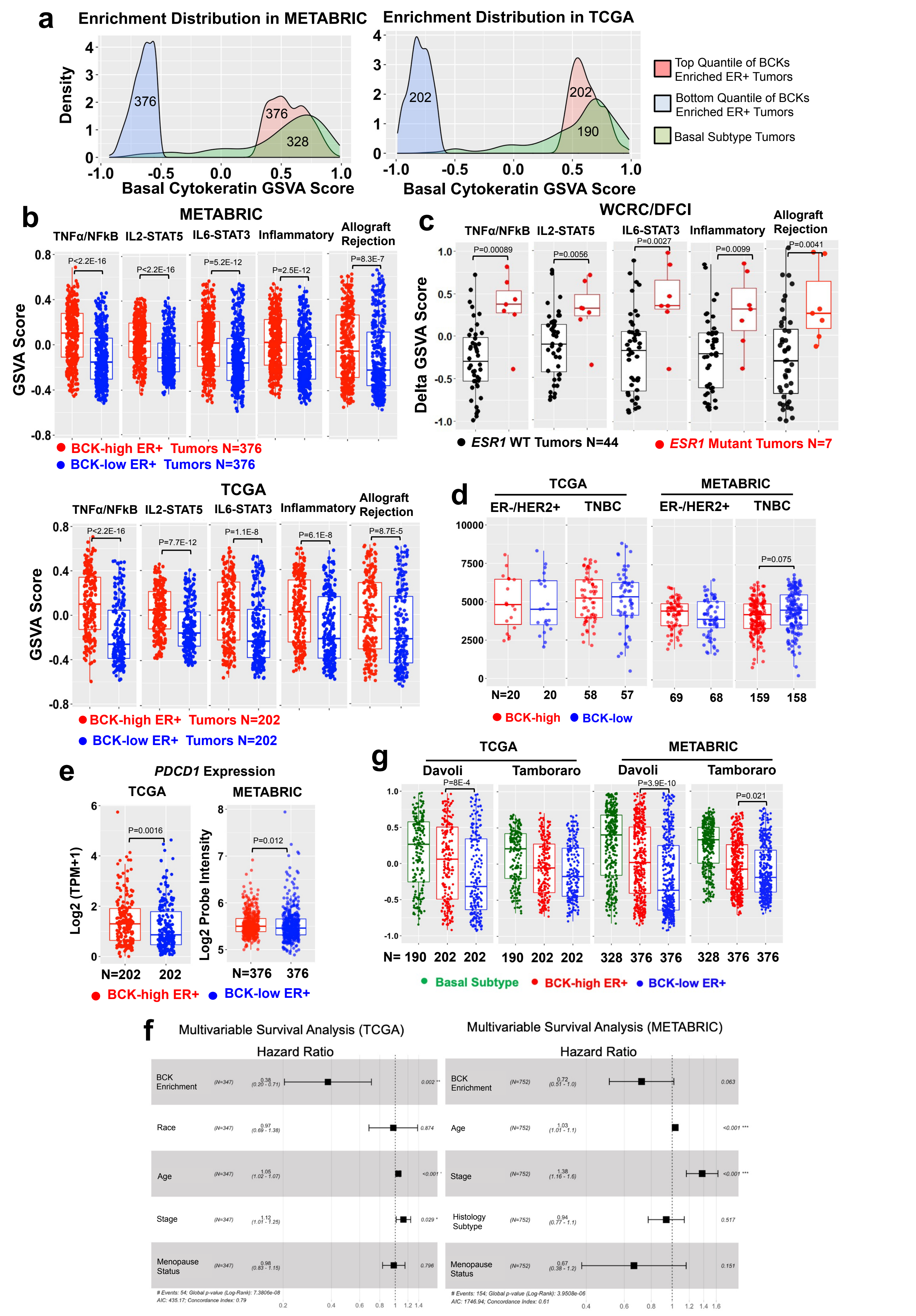

Supplementary Figure 12-Continued

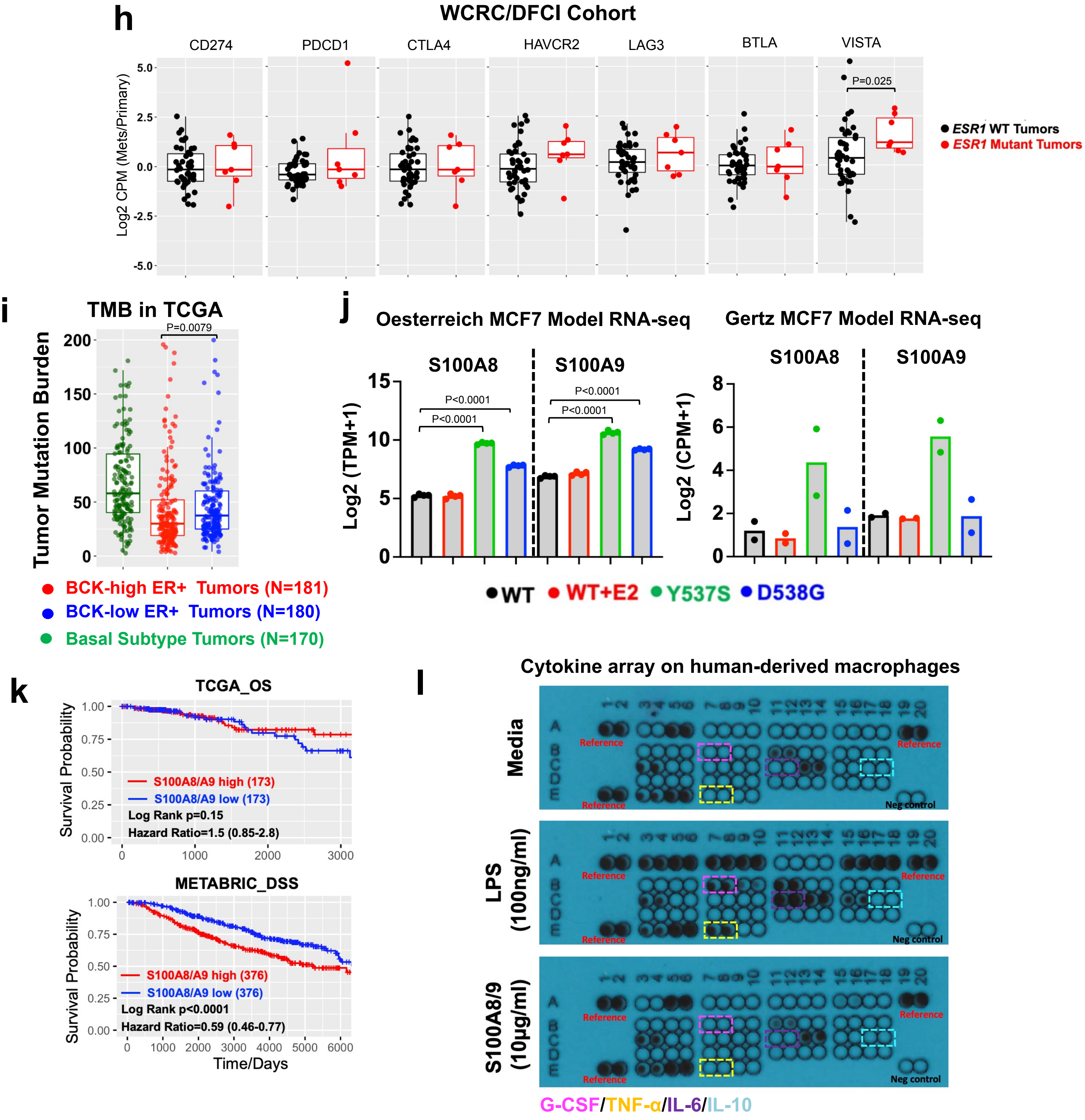

Supplementary Figure 12. Basal cytokeratins are associated with enhanced immune activation in ER+ tumors. (Related to Fig. 5 and 6)

a) Density plots showing the expressional distribution of BCKs GSVA scores in TCGA and METABRIC ER+ tumors for the bottom (blue) and top (red) quantile used for downstream analysis. BCKs enrichment in basal breast cancers (green) was set as control. Specific numbers of each subpopulation were indicated in the plots.

b) and c) Box plots showing the enrichment scores of the five intersected immune-related pathways between BCK-high (n=202 TCGA; n=376 METABRIC) and low (n=202 TCGA; n=376 METABRIC) subsets in ER+ tumors in METABRIC (b, Top panels) and TCGA (b, Lower panel), and between intra-patient primary tumor-paired *ESR1* mutant (n=7) and WT (n=44) metastatic lesions (c). Box plots span the upper quartile (upper limit), median (centre) and lower quartile (lower limit). Whiskers extend a maximum of 1.5X IQR. Mann Whitney U test (two-sided) was used for each comparison.

d) Dot plots showing the immune score based on ESTIMATE evaluations in BCK-high (n=20 and 57 for HER2+ and TNBC in TCGA; n=68 and 158 for HER2+ and TNBC in METABRIC) and low (n=20 and 58 for HER2+ and TNBC in TCGA; n=69 and 159 for HER2= and TNBC in METABRIC) subsets of TNBC and ER-/HER2+ tumors in TCGA and METABRIC cohorts. Specific sample numbers are labelled below each subpanel. BCK high and low subsets were separated based on median of GSVA score. Box plots span the upper quartile (upper limit), median (centre) and lower quartile (lower limit). Whiskers extend a maximum of 1.5X IQR. Mann Whitney U test (two-sided) was used.

e) Box blots representing *PDCD1* expression between BCKs-high (n=202 TCGA; n=376 METABRIC) and low (n=202 TCGA; n=376 METABRIC) ER+ tumors in TCGA and METABRIC. Log2 (TPM+1) from TCGA RNA-seq and log2 normalized probe intensity from METABRIC were used for plot. Box plots span the upper quartile (upper limit), median (centre) and lower quartile (lower limit). Whiskers extend a maximum of 1.5X IQR. Mann Whitney U test (two-sided) was used for each comparison.

f) Forest plot showing the hazard ratios of BCK enrichment levels together with other four key clinical features calculated in a multivariable survival analysis in TCGA (n=347) and METABRIC (n=752) ER+ tumors respectively. Data are presented as hazard ratio (dots) ± 95% CI (error bars). Log-rank test (two-sided) was applied to

compute the statistical significance of each factor within the Cox proportional hazard model. P values were labelled at the right side.
g) CD8 T cell enrichment score based on GSVA analysis of Davoli and Tamboraro signatures in basal tumors (METABRIC n=328; TCGA n=190), BCK-high (METABRIC n=376; TCGA n=202) and low (METABRIC n=376; TCGA n=202) subsets of ER+ tumors in TCGA and METABRIC. BCKs high and low were defined by the upper and bottom quartiles of each subset. Box plots span the upper quartile (upper limit), median (centre) and lower quartile (lower limit). Whiskers extend a maximum of 1.5X IQR. Mann Whitney U test (two-sided) was used for comparison.
h) Box plots representing the seven immune checkpoint gene expression in primary-matched paired metastatic tumor samples. Log2 (CPM+1) values were used for calculation. Expressional fold changes in each metastatic tumor were normalized to the matched primary tumor. Box plots span the upper quartile (upper limit), median (centre) and lower quartile (lower limit). Whiskers extend a maximum of 1.5X IQR. Mann-Whitney U test (two-sided) was performed to compare the expression between *ESR1* WT (N=44) or *ESR1* mutant (N=7) paired tumors.
i) Box plot showing comparison of tumor mutation burdens in TCGA basal subtype tumors (n=190) and BCKs high (n=202) and low (n=202) ER+ tumors. Tumor mutation burdens were calculated as 2X truncating mutation numbers + non-truncating mutation numbers of each tumor. Box plots span the upper quartile (upper limit), median (centre) and lower quartile (lower limit). Whiskers extend a maximum of 1.5X IQR. Mann Whitney U test (two-sided) was applied.
j) Bar graphic view of S100A8 and S100A9 gene expression based on RNA-seq data of Oesterreich (n=4, Log2 (TPM+1)) and Gertz (n=2, Log2 (CPM+1)) MCF7 *ESR1* mutant cells models. Dunnett's test was utilized for the Oesterreich model. (** p<0.01)
k) Kaplan-Meier plots showing the disease-specific survival (DSS) (METABRIC) and overall survival (OS) (TCGA) comparing patients with ER+ S100A8/A9 high vs low tumors. BCKs high and low were defined by the upper and bottom quartiles of each subset. Censored patients were labelled in cross symbols. Log-rank test (two-sided) was used and hazard ratio with 95% CI were labelled.
l) Supernatants from human-derived monocytes were treated with media alone, 100ng/ml LPS, or 10μg/ml S100A8/A9 for 24 hours and analyzed by the Proteome Profiler Human Cytokine Array Kit. Images of array membranes are shown. Negative

and positive control spots are labeled and increased cytokines are highlighted. This experiment was done once.

Source data are provided as a Source Data file for a-e, g-k.

Supplementary Figure 13

Supplementary Figure 5a

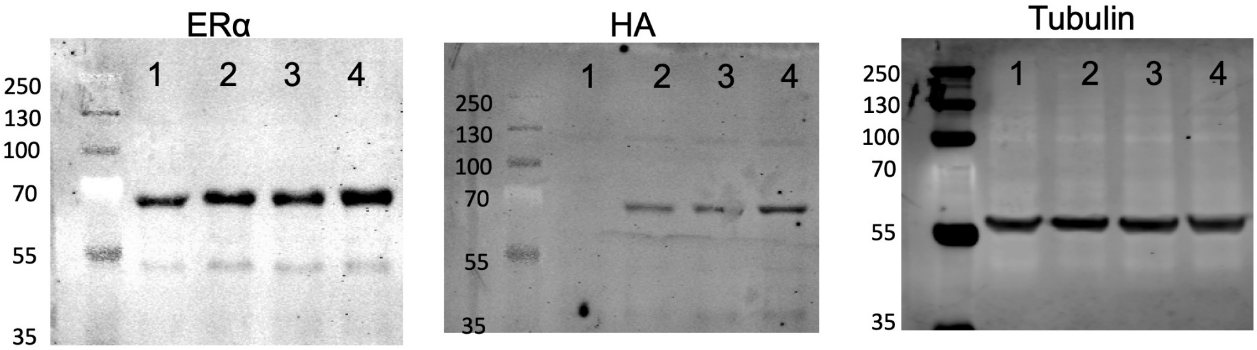

Lane1: pcDNA empty vector
Lane2: WT ESR1 Overexpressed MCF7
Lane3: Y537S ESR1 Overexpressed MCF7
Lane4: D538G ESR1 Overexpressed MCF7

Supplementary Figure 6a

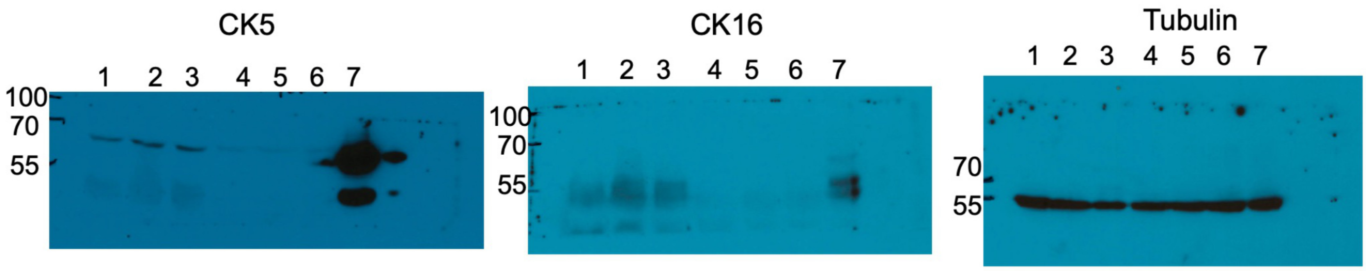

Lane1: MCF7 WT (Early Passage)
Lane2: MCF7 Y537S (Early Passage)
Lane3: MCF7 D538G (Early Passage)
Lane4: MCF7 WT (Late Passage)
Lane5: MCF7 Y537S (Late Passage)
Lane6: MCF7 D538G (Late Passage)
Lane7: MDA-MB-468

Supplementary Figure 10e

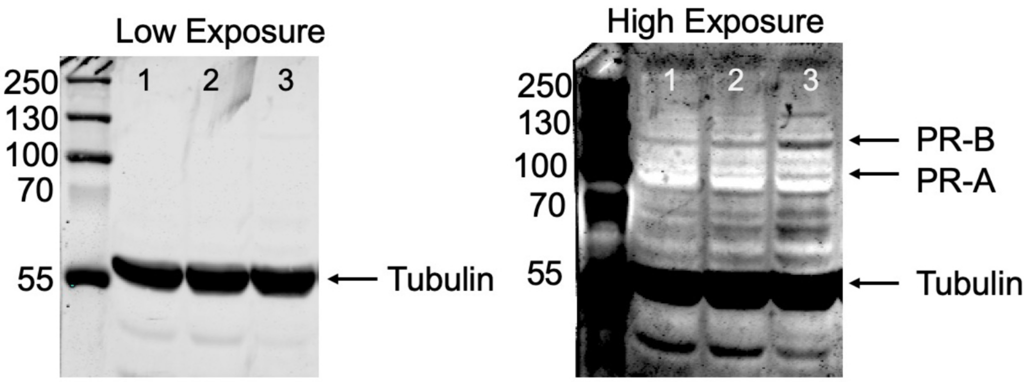

Lane1: MCF7-WT
Lane2: MCF7-Y537S
Lane3: MCF7-D538G

Supplementary Figure 13. Images of uncropped western blots provided in the Supplementary Information file.

Supplementary Table and Legends
Supplementary Table1

Pair ID	Primary Tumor ID	Metastatic Tumor ID	Cohort	Metastatic Site	*ESR1* Genotype	Mutation Variants
**1**	43P	43M	WCRC	Bone	Mutation	D538G
**2**	BP72	BM72	WCRC	Brain	Mutation	Y537C & L536Q
**3**	0031.50T	0006.56M	WCRC	Ovary	Mutation	Y537N
**4**	GP4	GM4B	WCRC	GI	Mutation	Y537S & D538G
**5**	043_Archival1	043_Prospective1	DFCI	Liver	Mutation	D538G
**6**	295_Archival1	295_T2	DFCI	Liver	Mutation	E380Q
**7**	325_Archival1	325_T1	DFCI	Liver	Mutation	D538G
**8**	19P	19M	WCRC	Bone	WT	
**9**	22P	22M	WCRC	Bone	WT	
**10**	31P	31M	WCRC	Bone	WT	
**11**	34P	34M	WCRC	Bone	WT	
**12**	44P	44M	WCRC	Bone	WT	
**13**	48P	48M	WCRC	Bone	WT	
**14**	55P	55M	WCRC	Bone	WT	
**15**	60P	60M	WCRC	Bone	WT	
**16**	A25P	A25M	WCRC	Bone	WT	
**17**	2P_RCS	2M_RCS	WCRC	Brain	WT	
**18**	3P_RCS	3M_RCS	WCRC	Brain	WT	
**19**	4P_RCS	4M_RCS	WCRC	Brain	WT	
**20**	6P_RCS	6M_RCS	WCRC	Brain	WT	
**21**	BP7	BM7	WCRC	Brain	WT	
**22**	BP17	BM17	WCRC	Brain	WT	
**23**	BP47	BM47	WCRC	Brain	WT	
**24**	BP51	BM51	WCRC	Brain	WT	
**25**	BP62	BM62	WCRC	Brain	WT	
**26**	0029.50T	0003.56M	WCRC	Ovary	WT	
**27**	0030.50T	0004.56M	WCRC	Ovary	WT	
**28**	0032.50T	0008.56M	WCRC	Ovary	WT	
**29**	0033.50T	0009.56M	WCRC	Ovary	WT	
**30**	0034.50T	0014.56M	WCRC	Ovary	WT	
**31**	0035.50T	0018.56M	WCRC	Ovary	WT	
**32**	OP1	OM1	WCRC	Ovary	WT	
**33**	OP5	OM5A	WCRC	Ovary	WT	
**34**	OP5	OM5B	WCRC	Ovary	WT	
**35**	OP8	OM8	WCRC	Ovary	WT	
**36**	GP2	GM2A	WCRC	GI	WT	
**37**	GP2	GM2B	WCRC	GI	WT	
**38**	GP4	GM4A	WCRC	GI	WT	
**39**	GP7	GM7	WCRC	GI	WT	
**40**	069_Archival1	069_Prospective1	DFCI	Bone	WT	
**41**	074_Archival1	074_Prospective1	DFCI	Liver	WT	
**42**	076_Archival1	076_Prospective1	DFCI	Liver	WT	
**43**	188_Archival1	188_Prospective1	DFCI	Bone	WT	
**44**	195_Archival1	195_Prospective1	DFCI	Liver	WT	
**45**	291_Archival1/ 291_Archival2	291_T1	DFCI	Liver	WT	
**46**	295_Archival1	295_Archival2	DFCI	Bone	WT	
**47**	306_Archival2	306_T1	DFCI	Breast	WT	
**48**	307_A1	307_T1	DFCI	Liver	WT	
**49**	348_A1	348_T1	DFCI	Liver	WT	
**50**	381_A1	381_T1	DFCI	Liver	WT	
**51**	495_A1	495_T1	DFCI	Liver	WT	

Supplementary Table1. Detailed intra-patient primary metastasis paired sample identities and *ESR1* mutation information from WCRC and DFCI cohort used in this study.

Supplementary Table 2

Rank	Gene	Log2FC (*ESR1* Mutant Tumor/WT Tumor)	Log2FC (MCF7 Y537S /MCF7 WT)	Log2FC (MCF7 D538G /MCF7 WT)
1	*KRT6B*	3.75	6.14	4.12
2	*KRT17*	3.27	5.84	3.59
3	*KRT16*	3.01	5.63	3.84
4	*S100A7*	0.48	7.03	4.07
5	*KRT6A*	2.30	5.78	3.35
6	*IFI27*	2.36	3.40	4.65
7	*KRT6C*	2.32	4.37	3.04
8	*SPRR1A*	0.67	5.84	3.16
9	*KRT5*	3.51	3.69	2.34
10	*PI3*	-0.03	6.21	2.89
11	*SLPI*	1.70	4.00	2.50
12	*S100A8*	1.07	4.46	2.54
13	*S100A9*	1.71	3.76	2.33
14	*SPRR1B*	0.25	5.12	2.22
15	*CALML5*	0.53	4.99	1.44
16	*DSG1*	1.43	3.42	2.03
17	*S100A2*	0.45	4.31	1.87
18	*FABP5*	0.83	3.34	2.40
19	*IFIT3*	0.70	2.43	3.01
20	*IFI44*	-0.37	2.91	3.59
21	*SBSN*	1.05	3.90	1.12
22	*KRT79*	0.08	4.74	1.20
23	*AKR1C2*	1.64	2.79	1.22
24	*SERPINB5*	0.75	3.29	1.39
25	*AMIGO2*	-0.01	2.58	2.63
26	*SAMD9*	-0.51	2.32	3.23
27	*GJA1*	-0.29	3.36	1.59
28	*KRT14*	3.68	0.55	0.41
29	*IVL*	0.46	3.17	0.85
30	*CTSC*	1.37	1.83	1.22
31	*TUBA4A*	1.18	2.09	1.13
32	*CEBPB*	2.08	1.47	0.83
33	*SLC7A5*	1.57	1.29	1.51
34	*LCN2*	0.33	3.19	0.69
35	*GAL*	1.02	1.24	1.90
36	*CALB2*	1.34	1.06	1.65
37	*SLC16A1*	0.09	1.86	1.91
38	*KLK6*	0.13	3.38	0.29
39	*C1R*	2.08	0.89	0.72
40	*UBD*	3.04	0.78	-0.23
41	*MT2A*	1.36	1.19	1.02
42	*TRIM29*	1.22	1.61	0.71
43	*RHCG*	0.94	2.29	0.20
44	*ANP32E*	1.60	0.67	1.00
45	*IL18*	0.44	1.69	1.10
46	*MT1X*	1.77	0.73	0.71
47	*CASP4*	1.12	0.88	1.19
48	*IGFBP6*	1.14	1.00	1.02
49	*LEMD1*	0.09	0.84	2.19
50	*PLSCR1*	0.30	1.25	1.53

Supplementary Table2. Top 50 consistently increased basal markers in MCF7 *ESR1* mutant cell models and clinical samples compared to WT counterparts. Six basal cytokeratins are highlighted in yellow.

Supplementary Table 3

Rank	Gene	Log2FC (T47D Y537S /T47D WT)	Log2FC (T47D D538G /T47D WT)
1	*WLS*	1.35	1.07
2	*HTRA1*	1.08	1.05
3	*PSAT1*	2.03	0.09
4	*IGF2BP2*	1.27	0.66
5	*AKR1C2*	0.46	1.10
6	*TUBB6*	0.76	0.67
7	*KIRREL*	0.73	0.69
8	*DMD*	1.16	0.26
9	*ATP1B3*	0.93	0.33
10	*FZD6*	0.26	0.98
11	*SFN*	0.34	0.90
12	*AKR1C1*	0.59	0.61
13	*ZDHHC2*	0.68	0.45
14	*AMD1*	0.46	0.67
15	*CORO1C*	0.67	0.44
16	*CLMP*	0.32	0.78
17	*STK17A*	0.41	0.63
18	*LARP6*	0.38	0.58
19	*PLS3*	0.38	0.52
20	*FAM83D*	0.28	0.61
21	*FSCN1*	0.59	0.27
22	*FERMT1*	0.31	0.54
23	*LAMB3*	0.80	0.03
24	*CXCL1*	0.83	0.00
25	*TKT*	0.21	0.62
26	*OSMR*	0.60	0.22
27	*CASP1*	0.71	0.10
28	*BMP1*	0.22	0.59
29	*ACTN1*	0.74	0.06
30	*ANXA2*	0.15	0.62
31	*OSBPL3*	0.15	0.60
32	*PGM2*	0.12	0.62
33	*GPSM2*	0.38	0.33
34	*LOX*	0.36	0.34
35	*MT2A*	0.57	0.09
36	*ANTXR1*	0.28	0.37
37	*CFL2*	0.51	0.13
38	*SLC1A3*	0.14	0.48
39	*LAMC2*	0.44	0.17
40	*ARNTL2*	0.58	0.02
41	*CXCL3*	0.54	0.06
42	*SH3D19*	0.34	0.24
43	*TWSG1*	0.26	0.32
44	*DGKA*	0.28	0.30
45	*S100A10*	0.20	0.33
46	*FBLIM1*	0.31	0.21
47	*AKR1C3*	0.17	0.35
48	*YBX1*	0.10	0.42
49	*TBC1D1*	0.32	0.19
50	*ADORA2B*	0.42	0.09

Supplementary Table3. Top 50 consistently increased basal markers in T47D *ESR1* mutant cell models compared to WT cells.

Supplementary Table 4

Groups	#reads	%mapped	# ER binding peaks ($p<10^{-5}$)
WT-Input	16856221	94.21	
WT-veh	23447868	85	125
WT-E2	16375519	89.15	12472
Y537S-Input	17529190	92.47	
Y537S-veh	20516994	90.35	657
Y537S-E2	20680234	89.81	1847
D538G-Input	20702360	82.44	
D538G-veh	20222723	87.33	1016
D538G-E2	20606853	91.4	5403

Supplementary Table 4. ER ChIP-seq mapping quality control of MCF7 ESR1 WT and mutant cells. P-value for each peak were calculated by MACS2 using a dynamic Poisson distribution to capture local biases in read background levels.

Supplementary Table 5

METABRIC BCKs High ER+ Tumors	TCGA BCKs High ER+ Tumors	WCRC/DFCI *ESR1* Mutant Tumors
EPITHELIAL_MESENCHYMAL_TRANSITION	EPITHELIAL_MESENCHYMAL_TRANSITION	INTERFERON_GAMMA_RESPONSE
APICAL_JUNCTION	MYOGENESIS	TNFA_SIGNALING_VIA_NFKB
KRAS_SIGNALING_UP	APICAL_JUNCTION	IL6_JAK_STAT3_SIGNALING
TNFA_SIGNALING_VIA_NFKB	TNFA_SIGNALING_VIA_NFKB	APOPTOSIS
ANGIOGENESIS	COAGULATION	ALLOGRAFT_REJECTION
MYOGENESIS	KRAS_SIGNALING_DN	REACTIVE_OXIGEN_SPECIES_PATHWAY
WNT_BETA_CATENIN_SIGNALING	KRAS_SIGNALING_UP	IL2_STAT5_SIGNALING
COAGULATION	WNT_BETA_CATENIN_SIGNALING	INFLAMMATORY_RESPONSE
UV_RESPONSE_DN	ANGIOGENESIS	COMPLEMENT
NOTCH_SIGNALING	NOTCH_SIGNALING	HYPOXIA
TGF_BETA_SIGNALING	P53_PATHWAY	P53_PATHWAY
HYPOXIA	HYPOXIA	
HEDGEHOG_SIGNALING	APICAL_SURFACE	
IL2_STAT5_SIGNALING	HEDGEHOG_SIGNALING	
INFLAMMATORY_RESPONSE	IL6_JAK_STAT3_SIGNALING	
IL6_JAK_STAT3_SIGNALING	INFLAMMATORY_RESPONSE	
APICAL_SURFACE	IL2_STAT5_SIGNALING	
P53_PATHWAY	ALLOGRAFT_REJECTION	
ALLOGRAFT_REJECTION	UV_RESPONSE_DN	
	XENOBIOTIC_METABOLISM	

Supplementary Table 5. Significantly enriched hallmark gene sets in BCK high vs low ER+ tumors in TCGA and METABRIC and ESR1 mutant vs WT tumors in WCRC/DFCI cohorts.

Supplementary Table 6

Sample Number	Sample ID	*ESR1* Genotype	Mutation Subtype	cfDNA MAF%
1	TP17-M767	Mutation	Y537C	0.18
2	TP17-M773	Mutation	D538G	0.44
3	TP17-M781	Mutation	D538G	0.24
4	TP17-M788	Mutation	D538G; Y537N	3; 1.5
5	TP17-M834	Mutation	Y537C	0.6
6	TP17-M835	Mutation	Y537C; D538G	0.33; 0.26
7	TP17-M840	Mutation	Y537C	0.7
8	TP17-M847	Mutation	D538G	0.45
9	TP18-M11	Mutation	D538G	17.7
10	TP18-M16	Mutation	D538G	0.75
11	TP18-M25	Mutation	D538G	0.22
12	TP17-M768	WT		
13	TP17-M841	WT		
14	TP17-M848	WT		
15	TP17-M888	WT		
16	TP18-M10	WT		
17	TP18-M9	WT		
18	TP18-M17	WT		

Supplementary Table 6. Detailed sample identities and *ESR1* mutation status identified by plasma screen from 18 patients with ER+ metastatic breast cancer.

Supplementary Table 7

Primers for qRT-PCR

**Gene**	**Forward**	**Reverse**
RPLP0	TAAACCCTGCGTGGCAATC	TTGTCTGCTCCCACAATGAAA
ESR1	CGCCCCATATTTTGAACACAG	ACGAGTATGGAGAGTGTCAGG
PGR	TCGATGCTAGAAAACTCCTTGG	GTCAACTCCAGGACCTCTTATG
CTCF	GCCATTCAAGTGTTCCATGTG	CTCATGTGCCTTTTCAGCTTG
KRT5	TCAATCCGAAGGCAGAAACG	TGCGGTATGTAACTTGCTGG
KRT6A	TCCCCTCAACCTGCAAATC	CCACTTTGTTTCCAGAACCTTG
KRT6B	AGCTGAGAAACATGCAGGAC	GCTTGCAGTTCAACCTTGTTC
KRT14	GAATCGTATCTCTGGAGCCTG	TGCCATCTCTTGCTTCTACTG
KRT16	GAAGTCCAAGTCTTCCCAGTC	GATATCCCCATGAGCCATTCC
KRT17	GGATCGGGTTAAGGGAAAGAG	AGGAGACATAGGCGAGAGG

Primers for ChIP-qPCR

**Binding Sites**	**Forward**	**Reverse**
CTCF_Peak1	ACTGTGGTTTCTCTGACGC	TGAACAAGAGCCTATAAAACCCC
CTCF_Peak5	AAAAGCTCTCGTGGGTTCC	AGCAGGGACGAAGTGAAAC

Supplementary Table 7. Sequence information primers used in this study.

Supplementary Table 8

Description	PMID	Source	Unique ID
TCGA RNA-seq	26209429	Gene Expression Omnibus	GSE62944
METABRIC Expression Matrix	22522925	Synapse	Syn1688369
Single-cell RNA-seq in two bilateral bone metastasos	This Study	Gene Expression Omnibus	GSE190772
Oesterreich MCF7/T47D ESR1 mutant cells RNA-seq	28535794	Gene Expression Omnibus	GSE89888
Oesterreich MCF7 ESR1 mutant cells ChIP-seq	This Study	Gene Expression Omnibus	GSE125117
Gertz MCF7/T47D ESR1 mutant cells RNA-seq/ChIP-seq	33184109	Gene Expression Omnibus	GSE148279
Ali MCF7 ESR1 mutant RNA-seq/ChIp-seq	27748765	Gene Expression Omnibus	GSE78286
Brown MCF7 ESR1 mutant RNA-seq/ChIP-seq	29438694	Gene Expression Omnibus	GSE94493
Shapiro T47D ESR1 mutant RNA-seq	30419347	Gene Expression Omnibus	GSE108304
MCF7 CTCF Knockdown RNA-seq	27638884	Gene Expression Omnibus	GSE85088
MCF7 CTCF ChIP-seq	22955616	ENCODE	ENCSR560BUE
MCF7 RAD21 ChIP-seq	29126249	ENCODE	ENCSR703TNG
MCF7 STAG1 ChIP-seq	20219941	Gene Expression Omnibus	GSE25021
MCF7 SMC1A ChIP-seq	27739523	Gene Expression Omnibus	GSE76893
MCF7 CTCF ChIP-PET	27625391	Gene Expression Omnibus	GSE72816
MCF7 Hi-C	31949157	Gene Expression Omnibus	GSE130916
MCF7 PR ChIP-seq with vehicle, R5020 and progestrone treatment	26153859	Gene Expression Omnibus	GSE68359
MCF7 GR ChIP-seq with dexamethasone treatment	27062924	Gene Expression Omnibus	GSE72249
MCF7 ATAC-seq	29867222	Gene Expression Omnibus	GSE102441
MCF7 H3K4me2 ChIP-seq	22955616	ENCODE	ENCSR875KOJ
MCF7 H3K4me3 ChIP-seq	29126249	ENCODE	ENCSR958MIB
MCF7 H3K9ac ChIP-seq	22955616	ENCODE	ENCSR056UBA
MCF7 H3K27ac ChIP-seq	29126249	ENCODE	ENCSR752UOD
T47D H3K27ac ChIP-seq	26051943	Gene Expression Omnibus	GSE69112
ZR75-30 H3K27ac ChIP-seq	26051943	Gene Expression Omnibus	GSE69112
MM231 H3K27ac ChIP-seq	26051943	Gene Expression Omnibus	GSE69112
MM468 H3K27ac ChIP-seq	26406377	Gene Expression Omnibus	GSE65201
HCC1954 H3K27ac ChIP-seq	22156296	Gene Expression Omnibus	GSE29069
TCGA ATAC-seq	30361341	Gemonic Data Commons	
MCF7 tamoxifen resistant cell model RNA-seq	32424275	Gene Expression Omnibus	GSE128458
MCF7 tamoxifen and fulvestrant resistant cell model RNA-seq	30472020	Gene Expression Omnibus	GSE104985
MCF7/T47D/ZR75-1/BT474 tamoxifen resistant cell model RNA-seq	30143015	Gene Expression Omnibus	GSE111151
MCF7 tamoxifen resistant cell model microarray	21233418	Gene Expression Omnibus	GSE26459
MCF7 tamoxifen resistant cell model RNA-seq	31644911	Gene Expression Omnibus	GSE106681
MCF7 fulvestrant resistant cell model RNA-seq	31949157	Gene Expression Omnibus	GSE118713
SUM44 tamoxifen resistant cell model microarray	18974135	Gene Expression Omnibus	GSE12708
MCF7/T47D/ZR75-1/HCC1428/SUM44 LTED cell model microarray	27246191	Gene Expression Omnibus	GSE75971
SUM44/MM134 LTED cell model microarray	30180878	Gene Expression Omnibus	GSE116744
MM134VI and SUM44 E2 treated microarray	24425047	Gene Expression Omnibus	GSE50693
ZR75-1 E2 treated microarray	23023562	Gene Expression Omnibus	GSE38132
BT474 E2 treated microarray	16606439	Gene Expression Omnibus	GSE3834

Supplementary Table 8. Sources of all the publicly available data used in this study.
