# Supplementary material for: ESR1 mutant breast cancers show elevated basal cytokeratins and immune activation
Source: Nat Commun. 2022 Apr 19;13:2011. doi: 10.1038/s41467-022-29498-9 (PMC9019037; doi:10.1038/s41467-022-29498-9)
Supplement: Supplementary file 4 — Description of Additional Supplementary Files [file 41467_2022_29498_MOESM4_ESM.pdf]

Title: Supplementary Data1:
Description: Five pairs of luminal and basal gene sets used in this study.

Title: Supplementary Data2:
Description: PAM50 identity and original source of 97 breast cancer cell lines transcriptomic data used in this study
